# Understanding the probiotic health benefits of *Bifidobacterium animalis* subsp. *lactis*, BB-12™

**DOI:** 10.3389/fmicb.2025.1605044

**Published:** 2025-07-02

**Authors:** Fergus W. J. Collins, Natalia I. Vera-Jiménez, Anja Wellejus

**Affiliations:** Human Health Research, Novonesis, Hørsholm, Denmark

**Keywords:** probiotic, *Bifidobacterium*, microbiome, gut health, colic

## Abstract

Probiotics have a longstanding association with human health, yet the mechanisms behind their benefits are often unclear. To understand the mode of action for the efficacy of a probiotic, it is important to take a broad overview of the interactions between the microbe, its environment, and the host. The BB-12 *Bifidobacterium animalis* subsp. *lactis* strain is one of the most documented probiotic strains on the market and has been shown to be effective in alleviating symptoms of a low defecation frequency and infant colic, among others. In this review, we examine the wide range of preclinical and clinical data available for the strain, to help elucidate some of its potential mechanisms of action. We describe the defence mechanisms developed by the strain to ensure gastrointestinal survival and transit, as well as the current knowledge on how BB-12 interacts with the host epithelial lining and cells of the immune system and the relationship between the strain and the gut microbiota. Collectively, the well documented clinical efficacies demonstrated by BB-12 are most likely not through one single mechanism, but through the collective direct and indirect effects the strain has on both its environment and the host.

## Introduction

Our understanding of the gut’s impact on human health and wellness is evolving rapidly, driven by the recent expansion of research in this field (Sa’ed et al., 2023). The gut microbiota is the complex community of microbes residing in the gastrointestinal (GI) tract and is intertwined with gut health (Khalil et al., 2024). While a distinct healthy microbiota is difficult to define for an individual, when in a balanced and symbiotic state, it contributes to maintaining health through mechanisms such as pathogen exclusion and maintenance of immune balance in the gut (Hou et al., 2022). Perturbations in the gut microbiota have been associated with a range of intestinal and extraintestinal disorders, such as irritable bowel disease, metabolic disorders and cardiovascular diseases (Chen et al., 2021). Given the intimate link between gut microbes and human health, the gut makes an attractive target for a range of health conditions, one such example being through the use of probiotics (Gul and Durante-Mangoni, 2024). Probiotics are defined as live microorganisms which when administered in adequate amounts confer a health benefit on the host (Hill et al., 2014). The interaction between the gut microbiota and the host is complex, often involving immune, endocrine and neurological systems (Kasarello et al., 2023). This complexity is also recognized in the relationship between a probiotic and the host, with individual strains having a range of potential impacts in the GI tract (Hitch et al., 2022). Understanding this complex balance is key when trying to identify how a probiotic may act to promote the health of the host.

Strains from the *Bifidobacterium* genus have long been utilised as probiotic microbes. They are key gut commensals across all life stages and have a history of safe use, demonstrated survivability in the GI tract and evidence of health-promoting benefits (Hidalgo-Cantabrana et al., 2018). Bifidobacteria are one of the early colonizers of the newborn gut, with their absence associated with a range of negative health outcomes in infants (Saturio et al., 2021). Levels of bifidobacteria are reduced in adults and the distribution of species differs significantly compared to infants (Arboleya et al., 2016). Strains of bifidobacteria have demonstrated the ability to alleviate symptoms in a range of health conditions, such as irritable bowel syndrome, atopic dermatitis, and metabolic syndrome in human clinical trials (Whorwell et al., 2006; Fang et al., 2022; Chu et al., 2023). Probiotic traits however can be strain specific and cannot be automatically attributed to the genus or species as a whole (McFarland et al., 2018). Therefore, the extensive study of individual strains of bifidobacteria is required to understand their probiotic potential.

The BB-12 *Bifidobacterium animalis* subsp. *lactis* strain (BB-12 is a trademark of Chr. Hansen A/S – occasionally referred to as “BB-12”) is one of the most studied probiotic strains from the *Bifidobacterium* genus (Jungersen et al., 2014). The strain was originally deposited in a dairy culture collection in 1983 and has since been incorporated into a range of probiotic supplements, infant formula and fermented dairy products. Initially identified as a strain of *B. bifidum*, it has since been reclassified firstly as *B. lactis*, and more recently as *B. animalis* subsp. *lactis* (Jungersen et al., 2014). The strain has been granted GRAS (generally recognized as safe) status in the United States, and the species has QPS (qualified presumption of safety) status in the European Union. The safety of the strain has also been demonstrated across populations in numerous clinical studies and the complete genome has been sequenced and is publicly available in NCBI GenBank under accession number CP001853.2 (Tan et al., 2017; Merenstein et al., 2015; Luoto et al., 2010; Garrigues et al., 2010; Jensen et al., 2021). Over time, the BB-12 strain has been used under different strain designations, such as LKM512, JCM 10602 and VTT E-012010 (Matsumoto, 2000; Ambalam et al., 2014; Saarela et al., 2005). For the purpose of this review, the strain will be referred to exclusively as BB-12.

**Figure 1:**
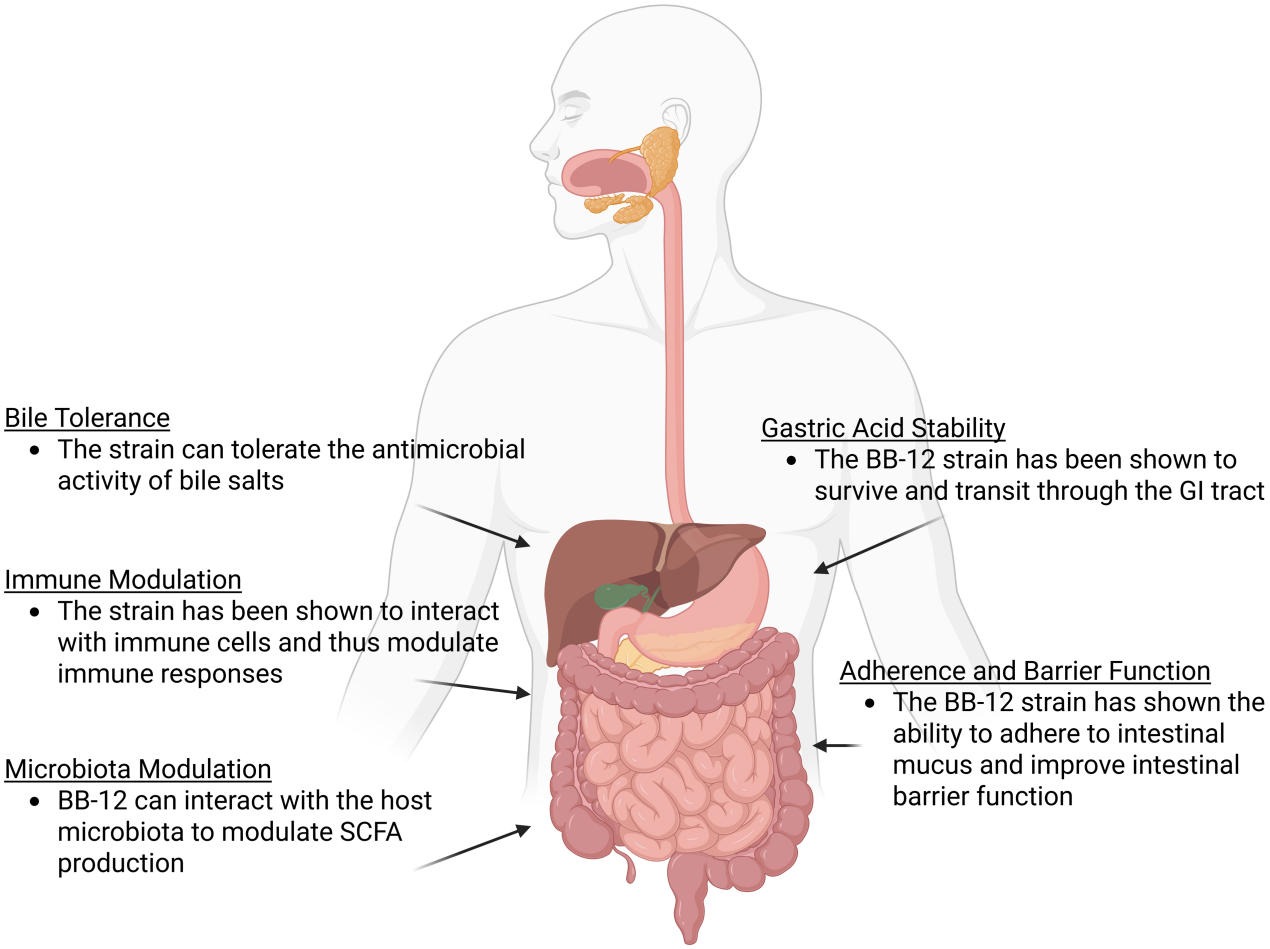
An overview of some of the potential probiotic mechanisms of action demonstrated by the *B. animalis* subsp. *lactis* BB-12 strain as outlined in this review. Figure created in https://BioRender.com.

The BB-12 strain has been studied extensively in both preclinical and clinical trials and has been documented in over 400 scientific publications. Our goal in this study is to consolidate and review this data with an aim of elucidating the mechanisms of actions by which the strain can survive in the GI tract and impart health benefits to the host (Figure 1). This will focus on areas such as understanding how the strain survives in the acidic conditions of the gut and the antimicrobial activity of bile salts, key traits for maintaining probiotic viability in the GI tract (Schöpping et al., 2022). The adhesion of the strain to the epithelial lining and its impact on GI barrier function will be evaluated, as well as the effect of the strain on the host microbiota and host immune system. We will also evaluate a range of clinical trials involving the strain, focussing on the impact of the strain in infant colic and in individuals experiencing a low frequency of defecation, two critical areas where the strain has demonstrated benefits (Table 1). While the insights from this existing data allows us to understand potential mechanisms of action for the BB-12 strain, it also highlights areas where additional novel data could be generated for BB-12 allowing for further expansion on the extensive data already generated for the strain.

**Table 1:** Overview of human clinical trials involving supplementation of *B. animalis* subsp. *lactis* BB-12 referenced in this study.

References	Population (*n*)	Dose of *B. animalis* subsp. *lactis* BB-12	Duration of supplementation	Format	Additional probiotic or prebiotic ingredients in test product
Gastric stability and transit					
Saxelin et al. (2010)	Adults (*n =* 36)	1.80E+09 CFU/dose (capsule), 1.40E+10 CFU/dose (yoghurt), 4.20E+07–1.20E+06 CFU/dose (cheese)	2 weeks	Capsules, yoghurt, or cheese	*L. rhamnosus* GG, *L. rhamnosus* LC705 & *P. freudenreichii* subsp. *shermanii* JS
Dotterud et al. (2015)	Pregnant Women (*n =* 415)	5.00E+10 CFU/day	From 36 weeks of gestation to 3 months after birth of the child	Fermented milk	*L. rhamnosus* GG, *L. acidophilus* LA-5
Melsaether et al. (2023)	Mothers and infants. Infants supplemented directly or exposed through maternal supplementation (*n =* 47)	2.00E+09 CFU/day (mothers), 5.00E+08 CFU/day (infants)	From 33 weeks of gestation until 28 days postnatal for mothers, 28 days for infants	Capsule (mothers) or sachet (infants)	*B. infantis* Bifin02, *L. rhamnosus* GG, *L. acidophilus* LA-5 (mothers) and *B. infantis* Bifin02, *L. rhamnosus* GG (infants)
Mättö et al. (2006)	Adults (*n =* 14)	1.00E+11 CFU/day	10 days	Fermented milk	*L. acidophilus* NCFB 1748, *L. paracasei* subsp. *paracasei* LMG P-17806
Poutsiaka et al. (2017)	Adults (*n =* 27)	1.00E+09 CFU/day	3 weeks	Sachet	*L. rhamnosus* GG
Christensen et al. (2006)	Adults (*n =* 71)	1.00E+08, 1.00E+09, 1.00E+10 or 1.00E+11 CFU/day	3 weeks	Capsule	*L. paracasei* subsp. *paracasei* CRL-431
Bile tolerance					
Tonucci et al. (2017)	Adults with Type 2 Diabetes (*n =* 45)	1.00E+09 CFU/day	6 weeks	Fermented milk	*L. acidophilus* LA-5
Mohamadshahi et al. (2014)	Adults with Type 2 Diabetes (*n =* 44)	1.11E+09 CFU/day	8 weeks	Yoghurt	*L. acidophilus* LA-5
Chiu et al. (2021)	Adults with mild hypercholesterolemia (*n =* 40)	1.20E+08 CFU/day	10 weeks	Milk based formula	*L. acidophilus* LA-5, *L. casei* TMC
Lee et al. (2017)	Adults (*n =* 30)	3.16E+09 CFU/day	Crossover study with 4-week treatment arms, followed by 2-week washout periods	Capsule or yoghurt smoothie	-
Şahin et al. (2022)	Adults with newly diagnosed type 2 diabetes or prediabetes (*n =* 156)	NA	3 months	NA	-
Adherence and barrier function					
Krumbeck et al. (2018)	Adults (*n =* 94)	1.00E+09 CFU/day	3 weeks	Sachet	-
Immune system					
Meng et al. (2017)	Adults (*n =* 30) (same study population as Lee et al., 2017)	3.16E+09 CFU/day	Crossover study with 4-week treatment arms, followed by 2-week washout periods	Capsule or yoghurt smoothie	-
Rizzardini et al. (2012)	Adults (*n =* 211)	1.00E+09 CFU/day	6 weeks	Capsule	-
Merenstein et al. (2015)	Adults on antibiotics (*n =* 40)	1.00E+10 CFU/day	10 days	Yoghurt	-
Ringel-Kulka et al. (2015)	Children 12–48 months old (*n =* 149)	5.00E+09 CFU/day	16 weeks	Yoghurt	*S. thermophilus*, *L. bulgaricus*, Inulin
Gut microbiota					
Matsumoto et al. (2007)	Adults with atopic dermatitis (*n =* 10)	5.20E+09 CFU/day	4 weeks	Yoghurt	-
Matsumoto and Benno (2004)	Adults (*n =* 7)	5.20E+09 CFU/day	2 weeks	Yoghurt	-
Matsumoto et al. (2001)	Elderly adults (*n =* 6)	NA	2 weeks	Yoghurt	-
Manzoni et al. (2017)	Elderly adults (*n =* 29)	1.50E+10 CFU/day	4 weeks	Beverage	-
Matsumoto et al. (2017)	Adults (*n =* 27)	6.00E+09 CFU/day	12 weeks	Sachet	-
Merenstein et al. (2021)	Adults (*n =* 62)	1.00E+10 CFU/day	2 weeks	Yoghurt	-
Low defecation frequency					
Uchida et al. (2005)	Women (*n =* 41)	1.00E+09 CFU/day	2 weeks	Fermented milk	-
Uchida et al. (2004)	Women (*n =* 38)	1.00E+08 CFU/day	2 weeks	Fermented milk	-
Eskesen et al. (2015)	Adults with low defecation frequency and abdominal discomfort (*n =* 1,248)	1.00E+09 or 1.00E+10 CFU/day	4 weeks	Capsule	-
Pitkälä et al. (2007)	Elderly nursing home residents (*n =* 209)	1.00E+09 CFU/day	7 months	Fermented oat drink	-
Colic					
Nocerino et al. (2020)	Infants ≤7 weeks of age with infant colic (*n =* 80)	1.00E+09 CFU/day	4 weeks	Oil suspension	-
Chen et al. (2021)	Infants ≤3 months of age with infant colic (*n =* 192)	1.00E+09 CFU/day	3 weeks	Oil suspension	-

## Gastric stability and transit

The ability of a bacterial strain to tolerate acid stress can be an important trait for a probiotic, allowing it to survive the low gastric pH after ingestion and to tolerate organic acids produced by microbes in the GI tract (Schöpping et al., 2022). The low gastric pH in the stomach, as well as the activity of gastric enzymes such as pepsin, combined with the ionic strength of the environment may impact the viability of microbes, forming a barrier helping to prevent ingested microbes from translocating across the intestinal tissue and into the bloodstream (Han et al., 2021). The greater the tolerance of a probiotic to the gastric environment, the higher the number of viable bacterial cells will enter the intestine. As well as hydrochloric acid present in the gastric juice, the organic acids produced by microbes in the GI tract may also inhibit bacterial growth. Here the weak acids can enter bacterial cells where they dissociate, releasing a proton and thus decreasing the intracellular pH of the microbe (Guan and Liu, 2020).

**Figure 2:**
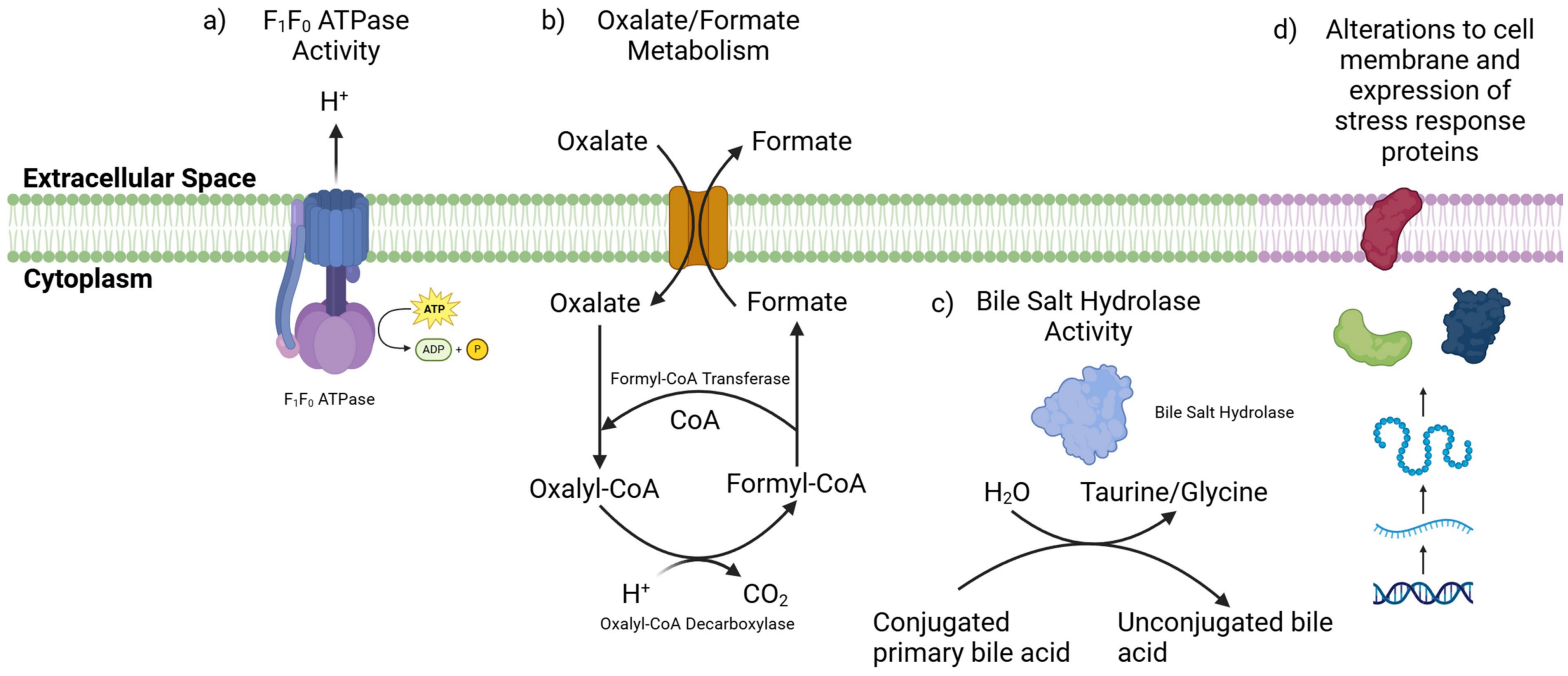
Mechanisms by which the BB-12 strain may tolerate acid and bile stress. (a) The F_1_F_0_ ATPase pump can export H + ions from the cytoplasm, reducing internal acidification of the cell. (b) The import of oxalate and its conversion to formate consumes an H + ion in cells. (c) Bile salt hydrolase enzymes can deconjugate primary bile acids which impacts their biochemical properties. (d) An upregulation of stress response proteins and alterations in the cell membrane composition may also act to protect the BB-12 strain from the antimicrobial activity of bile acids. Figure created in https://BioRender.com.

Numerous mechanisms have been outlined by which bifidobacteria may respond to these stresses associated with survival and transit in the GI tract (Figure 2) (Schöpping et al., 2022). One mechanism through which BB-12 can tolerate low gastric pH is through the activity of an F_1_F_0_-ATPase (Matsumoto et al., 2004). F_1_F_0_-ATPase is a membrane bound transporter which couples ATP synthesis and degradation to the movement of protons across cell membranes (Sun, 2016). In respiring bacteria, the proton motive force across the membrane of the cell is used to generate ATP through the activity of the F_1_F_0_-ATPase. In bifidobacteria, which lack a respiratory chain, this is reversed and the F_1_F_0_-ATPase can pump H + ions out from the cytoplasm of the cell by hydrolysing ATP thus reducing internal acidification (Ventura et al., 2004).

The activity of the F_1_F_0_-ATPase has been demonstrated to be an important mechanism for the acid tolerance of many lactic acid bacteria, and transcription of the genes for this complex is upregulated in response to low pH (Ventura et al., 2004). In a study with BB-12, the F_1_F_0_-ATPase activity of the strain was found to increase upon exposure to lower pHs. This increased activity demonstrates the ability of the BB-12 strain to extrude excess protons from the cytoplasm of the cells under acidic conditions and thereby enhance survival. The F_1_F_0_-ATPase activity in less acid tolerant strains did not show this increase in activity in response to lower pHs, indicating that these strains were not able to adapt optimally to the acidified environment (Matsumoto et al., 2004).

Bacterial strains also have the capacity to alleviate acid stress through metabolic activities, for example by the conversion of oxalate to formate (Schöpping et al., 2022). The formyl-CoA transferase (EC 2.8.3.16) and oxalyl-CoA decarboxylase (EC 4.1.1.8) enzymes encoded by the BB-12 strain are both involved in this process. Here, the intracellular conversion of oxalate to formate results in the consumption of one intracellular proton and upon secretion of formate from the cell, the internal acidification will be reduced (Schöpping et al., 2022). The ability of the BB-12 strain to metabolize oxalate has previously been demonstrated *in vitro* (Turroni et al., 2010). Another strain of *B. animalis* subsp. *lactis* showed increased oxalate metabolism at lower pH, identifying this process as a response to acid stress in strains of *B. animalis* subsp. *lactis*. This ability to metabolize oxalate was also demonstrated in an *in vivo* mouse model using the *B. animalis* subsp. *lactis* DSM 10140 strain, which was shown to degrade dietary oxalate and thus limit its absorption in the intestine. This activity could potentially play a role in reducing the systemic oxalate levels which can increase the risk of hyperoxaluria, a risk factor for the development of calcium oxalate kidney stones (Klimesova et al., 2015).

Acid tolerance, as well as successful GI transit, has been extensively demonstrated for the BB-12 strain in various model systems. In *in vitro* assays, BB-12 has shown survival rates of up to 86% after exposure to artificial gastric juice at pH 2.5 for 3 h (Liu et al., 2020). The high acid tolerance of the BB-12 strain was further substantiated in a study where the survival rate of BB-12 exposed to a pH range of 2–4 for 20 min was much higher compared to strains of the *B. longum*, *B. infantis* and *B. adolescentis* species (Vernazza et al., 2006). To better simulate the human GI tract, the survival of the BB-12 strain was also evaluated in simulated oral, gastric and intestinal phases *in vitro*. In all compartments of the simulated environment, BB-12 demonstrated high survival, highlighting the potential of the strain to survive effectively in the GI tract (Kim et al., 2022).

Several animal models have also demonstrated the effective transit of the BB-12 strain through the GI tract. Following dosing of germ free mice with 1E+08 CFU of BB-12, the isolation of the strain at high numbers in faeces after 1 day of feeding demonstrated its effective survival, growth and transit through the murine GI tract (Martins et al., 2009). In a gnotobiotic preterm piglet model, the strain was also found to be present and viable in the jejunum, ileum and colon, outlining the adaptation of the strain to the gastric environment (Splichalova et al., 2021). Interestingly, the BB-12 strain was also shown to transit and proliferate in the canine GI tract which demonstrates a high degree of acid tolerance for the strain, as the canine gastric pH remains acidic during meal consumption unlike in humans where the pH is buffered by food (Nakamura et al., 2015; Mahar et al., 2012).

The ability of the BB-12 strain to survive and transit through the human GI tract has been demonstrated across numerous human clinical trials using different application formats, such as capsules, sachets and fermented dairy products. The strain has been shown to transit in a range of populations, such as healthy adults, pregnant women, infants and obese adults (Saxelin et al., 2010; Dotterud et al., 2015; Melsaether et al., 2023; Krumbeck et al., 2018). Studies have also shown that transit of the BB-12 strain can be identified quite early into the supplementation periods, for example, using a RAPD PCR technique, Mättö et al. (2006) were able to identify BB-12 in the stool of 11 out of 14 participants after 10 days of supplementation with a yoghurt containing the strain. While some of these detection techniques involve qPCR which relies on the presence of DNA from the strain, and does not necessarily distinguish between live and dead microbes, one study was carried out which correlated the detection of BB-12 by qPCR in stool samples with viable cell counts, demonstrating that the strain was transiting through the GI tract alive (Poutsiaka et al., 2017).

In a study aimed at understanding the impact of the format of supplementation on transit, the BB-12 strain was administered in the form of a capsule, a yoghurt or in cheese (Saxelin et al., 2010). BB-12 was identified in the stool of all participants thus outlining the effective transit of the strain. The results appeared to indicate that the faecal load of BB-12 was significantly higher in the individuals supplemented with yoghurt compared to those supplemented with capsules or cheese, however, these differences were likely related to the higher intake of the strain in yoghurt (1.40E+10 CFU/day) compared to that in the capsule (1.80E+09 CFU/day) or cheese (1.60E+06–4.2E+07 CFU/day). This dose–response has also been seen in another study where increased concentrations of the strain supplemented in capsules corresponded to greater numbers of cells isolated in the faeces (Christensen et al., 2006). The dose of the strain may therefore have a greater impact than the supplement format on the recovery of BB-12 in faecal samples.

While these studies demonstrate the effectiveness of the BB-12 strain in surviving and transiting effectively in the GI tract, many also evaluate the ability of the strain to colonize the GI tract after supplementation has ended. In one study, the persistence of the strain was found to differ between supplementation formats, with the individuals supplemented with BB-12 in a yoghurt matrix demonstrating a longer persistence compared to those receiving capsules or cheese, however, this likely again relates to the higher dose of the strain in the yoghurts. Despite the longer persistence of BB-12 in those receiving yoghurt, by the end of the 21-day washout period only 3 of 36 participants had detectable levels of BB-12 in their stool. This suggests that the strain is a transient colonizer in humans and does not colonize the gut long-term (Saxelin et al., 2010). In an open label trial where infants were directly fed a probiotic mixture containing BB-12, the positive detection of BB-12 in faecal samples dropped from 100% of infants during supplementation to 35% after a 2-week washout period. In the infants with detectable BB-12 in faeces after the washout period, the large drop in the abundance of the strain suggests that BB-12 is being washed out from the gut, and testing after a further washout period could potentially have confirmed the lack of colonization of the strain in these infants (Melsaether et al., 2023).

Taken together, this data demonstrates how the BB-12 strain has adapted to survive human gastric conditions and transit effectively through the gastro-intestinal tract. This ability of BB-12 to survive gastrointestinal transit increases the potential for the strain to proliferate and be metabolically active in the gut where it may then exert beneficial probiotic effects.

## Bile tolerance

Another important hurdle microbes must overcome to survive in the GI tract is bile stress. Bile is synthesized in the liver, stored in the gallbladder and has a crucial function in the GI tract by enabling absorption of dietary lipids (Begley et al., 2005). One of the major components of bile are bile salts, which are produced by hepatocytes through the metabolism of cholesterol. These primary bile salts are produced from cholic acid or chenodeoxycholic acid, which are then conjugated with the amino acids taurine or glycine (Schubert et al., 2017). This conjugation reaction lowers the p*K*_a_ of the bile salts, thus improving their hydrophilicity leading to better water solubility (Begley et al., 2005). Most (approximately 95%) of the bile salts released into the intestine are reabsorbed by the time they reach the terminal ileum and are transported back to the liver. The remaining 5% is mostly excreted in the faeces and this net loss of bile salts is replaced by *de novo* bile salt synthesis from cholesterol in the liver (JG Marin et al., 2016). Bile salts display antimicrobial activity, as their amphipathic nature allows them to target and solubilize bacterial cell membranes which causes cell membrane leakage, cytoplasm acidification and eventually cell death. Additionally, bile salts can damage DNA and proteins in bacterial cells as well as cause oxidative stress which can all be detrimental to the bacteria (Begley et al., 2005).

Certain gut microbes have developed specific defence mechanisms against the activity of bile salts, the production of bile salt hydrolase (BSH) enzymes is one such mechanism. These enzymes can deconjugate primary and secondary bile salts by removing the glycine or taurine residue from molecules through hydrolysis of an amide bond (Bourgin et al., 2021). Oxidation, epimerization, desulfation and/or esterification of the deconjugated bile acids by the microbiota can then result in the formation of secondary bile acids (Collins et al., 2023). Recently, it was discovered that certain bacteria also hold the capacity to conjugate additional amino acids to the deconjugated bile acid backbone, thus increasing the diversity in the pool of potential bile acids in the GI tract (Quinn et al., 2020).

The BB-12 strain has been shown to survive and grow in the presence of a range of bile salts *in vitro*, with several potential mechanisms identified by which the strain may survive bile exposure (Figure 2). Vinderola and co-workers found that the BB-12 strain both grows on and deconjugates tauro-deoxycholate and glyco-deoxycholate salts and also can grow in the presence of taurocholate and glycocholate salts (Vinderola and Reinheimer, 2003). This study further outlines the ability of the strain to grow in media supplemented with 0.3, 0.5, and 1% bile (Vinderola and Reinheimer, 2003). The high survival rate of the strain in simulated intestinal conditions containing bile extract also highlights the ability of the strain to tolerate bile stress (Kim et al., 2022).

A bacterial transcriptomics study identified a significant change in expression of over 200 genes when the BB-12 strain was grown in 0.1% bile (Garrigues et al., 2005). The functions of the pathways impacted varied greatly in the strain. For example, the down-regulation of genes involved in fatty acid biosynthesis was observed, which could suggest that the cell membranes of BB-12 were impacted in response to bile exposure (Garrigues et al., 2005). Changes in cell membrane composition are hypothesized to play a role in helping to reduce membrane susceptibility to the antimicrobial activity of bile salts (Schöpping et al., 2022). There was also an upregulation of genes involved in general stress responses, such as the *dnaK* operon and the *groEL* gene after bile exposure (Garrigues et al., 2005). The upregulation of such genes can play a role in protecting the cells from damage induced by exposure to bile salts (Ruiz et al., 2013; Candela et al., 2010). Previously, the *B. animalis* subsp. *lactis* BI07 strain also demonstrated upregulation of surface receptors such as DnaK after exposure to bile. DnaK upregulation was associated with increased binding to plasminogen, a glycoprotein which is found in the intestine of the host (Candela et al., 2010). This suggests that bile exposure may act as a signal to strains of *B. animalis* subsp. *lactis*, inducing changes which can help these cells adapt to conditions in the GI tract, such as increasing their ability to adhere to intestinal cells (Schöpping et al., 2022; Candela et al., 2010).

The BB-12 strain also encodes a BSH enzyme (EC: 3.5.1.24) and has demonstrated both growth on, and deconjugation of, tauro-deoxycholate and glyco-deoxycholate (Vinderola and Reinheimer, 2003). The activity of BSH enzymes is thought to play an important role in alleviating the toxicity of the primary bile salts through deconjugation. The protective role of these deconjugation reactions is however debated, as in some instances the deconjugated bile acids can display a greater level of antimicrobial activity compared to the primary bile salts from which they were derived (Ruiz et al., 2013; De Smet et al., 1995). The deconjugated bile acids can act as weak acids and diffuse into the cell where, similarly to organic acids, they can release a proton leading to the acidification of the cytoplasm of the cell (Schöpping et al., 2022; Kurdi et al., 2003). To overcome this acid stress, BB-12 can utilize the same mechanisms as used for protection against gastric acid, such as through the activity of F_1_-F_0_-ATPases. The conversion of oxalate to formate can also remove additional protons from inside the cell (Schöpping et al., 2022; Sánchez et al., 2006). This potential resistance to the antimicrobial effects of deconjugated bile acids was previously demonstrated in another strain of *B. animalis* subsp. *lactis* (Grill et al., 2000). Given the ability of BB-12 to deconjugate primary bile salts and grow in the presence of these deconjugated bile acids, it is likely the strain also demonstrates similar effects (Vinderola and Reinheimer, 2003).

The ability of strains to generate these antimicrobial deconjugated bile acids through the activity of BSH enzymes could provide a mechanism by which these strains could inhibit the growth of competitors (Guzior and Quinn, 2021). The amino acids released by deconjugation reactions can also be used as a nutrient source for the BSH encoding microbes (Guzior and Quinn, 2021). For the BB-12 strain it was previously reported that the encoded BSH gene was constitutively expressed, which could provide a competitive advantage for the strain by allowing it to respond immediately to the presence of bile salts in the GI tract when ingested (Garrigues et al., 2005). The microbial conversion of bile acids can reduce their rate of reabsorption in the intestine, leading to higher levels of faecal secretion, which is associated with an increase in *de novo* synthesis of bile salts from cholesterol in the host (Kriaa et al., 2019).

This ability of the BB-12 strain to increase the excretion of bile acids was demonstrated *in vivo*, where rats ingesting BB-12 included in buffalo- or soy-milk yoghurt displayed greater levels of bile acid excretion in faeces compared to rats on control diets (Abd El-Gawad et al., 2005). The increased utilization of cholesterol for bile synthesis to replace the excreted bile acids can consequently reduce cholesterol levels in extrahepatic tissues and macrophages, and thereby potentially protect the host from cardiometabolic health risks (Chiang et al., 2020). Furthermore, the BB-12 strain was shown to reduce cholesterol levels in *in vitro* cultivation assays, where the strain was shown to assimilate cholesterol into cell membranes and bind it to cell wall peptidoglycans (Alhaj et al., 2010).

In human clinical studies, supplementation with yoghurt containing the BB-12 strain combined with the *Lactobacillus acidophilus* LA-5 strain was associated with improvements in cholesterol levels, an effect which appears improved over standard yoghurt controls (Tonucci et al., 2017; Mohamadshahi et al., 2014). A similar improvement in cholesterol markers was seen for another multi-strain formulation containing BB-12 in a milk based formula (Chiu et al., 2021). Given these improvements were seen in multi-strain formulations, it is difficult to elucidate the impact which BB-12 alone had in these cohorts. In the clinical studies where individuals were supplemented with the BB-12 strain alone, no impact was seen on the cholesterol levels of young healthy adults or in newly diagnosed individuals with type 2 diabetes or pre-diabetes (Lee et al., 2017; Şahin et al., 2022). In both of these studies, however, the average cholesterol levels of participants were within a healthy range (Aranmolate and Obayemi, 2018), making additional improvement difficult to achieve. Further work in populations with high cholesterol levels is warranted to fully understand the potential impact of BB-12 supplementation on cholesterol levels in individuals.

## Adherence and barrier function

In the GI tract, bacteria have the capacity to interact directly with host intestinal epithelial cells which form the primary barrier preventing microbes from entering the bloodstream (Yan and Polk, 2020). This ability to interact with host cells can provide a competitive advantage to microbes. For example, the mucus layer on the surface of the intestinal epithelium can act as a feed source for microbes and can form an environment for colonization. This mucus layer also acts as a physical barrier that helps reduce unwanted microbial interaction with epithelial cells (Han and Vaishnava, 2023). These microbial interactions can have a large impact on host cells and have been shown to impact intestinal barrier function, for example, through the upregulation of genes encoding tight junction proteins (Gou et al., 2022). Tight junctions are complexes of proteins found in the gaps between epithelial cells which connect neighboring cells and control paracellular flow of substances from the gut lumen into the bloodstream (Gou et al., 2022).

The ability of the BB-12 strain to interact with and adhere to intestinal epithelial cells has been evaluated in several studies and the strain has demonstrated varying degrees of adhesion to Caco-2 cells (Kim et al., 2022; Fernández de Palencia et al., 2008; Laparra and Sanz, 2009). Caco-2 cells are commonly used as a model of the intestinal epithelium; however, these lack a mucus layer. The combination of Caco-2 cells with mucus producing HT29-MTX cells therefore form a more accurate model of conditions in the intestine (Hoffmann et al., 2021). When tested on a co-culture of Caco-2/HT29-MTX cells, BB-12 and other probiotic strains demonstrated a lower level of adhesion compared to when co-cultured with Caco-2 cells alone (Laparra and Sanz, 2009). This suggests mucus production by HT29-MTX cells reduces bacterial cell adhesion. Several *ex vivo* studies have also been carried out with the BB-12 strain to determine its capacity to adhere to mucus. Co-incubation of the strain with mucus isolated from faeces of healthy individuals from a broad age range demonstrates the ability of the strain to adhere to human intestinal mucus, with stronger adhesion to adult mucus samples compared to infant mucus samples being identified (Harata et al., 2021; Matsumoto et al., 2002). This difference in the mucus binding capacity of the strain between HT29-MTX cells and human faecal mucus could be explained by differences in the types of mucus present in these assays. HT29-MTX cells, while originally isolated from a human colon, predominantly produce MUC5 mucin which is more typically found in the upper GI tract. Mucin isolated from faeces (MUC2 mucin) is however more common in the colon (Laparra and Sanz, 2009; Kitamura et al., 1996; Fancy et al., 2024). This indicates that BB-12 may have a greater capacity to bind to mucus in the large intestine compared to the upper GI tract which was demonstrated when the strain was found to show increased adherence to Caco-2 cells combined with type II porcine mucin compared to the Caco-2/HT29-MTX co-culture (Laparra and Sanz, 2009).

A study of the extracellular proteome of the BB-12 strain identifies nine protein homologs which can be important for adhesion of the strain (Gilad et al., 2011). Two of these proteins, GROEL and EF-TU, play important roles in protein synthesis and folding in the cell. Both proteins, however, have also demonstrated the capacity to bind different types of mucins in other bacterial species (Caldas et al., 1998; Bergonzelli et al., 2006; Granato et al., 2004). Several other proteins produced by BB-12 were also identified that promote adhesion to collagen and fibronectin, which has previously been demonstrated by the strain *in vitro* (Gilad et al., 2011; Ouwehand et al., 2004).

The BB-12 strain has shown in several *in vitro* studies the ability to improve epithelial barrier function through its interaction with intestinal epithelial cells. Co-incubation of the strain with Caco-2 cells was found to increase the transepithelial electrical resistance (TEER) of the cell line, suggesting strengthened barrier integrity. Both live and heat-inactivated versions of the BB-12 strain were evaluated in this assay, and while both were found to increase TEER, the live strain performed better, indicating that metabolites produced by the strain impacted the barrier function of intestinal cells (Castro-Herrera et al., 2020). These findings were supported by another study where cell free supernatants of the BB-12 strain grown on a variety of carbohydrate sources were found to significantly improve the TEER in Caco-2 cells compared to control treatments. This improvement in barrier function is thought to be related to the production of short chain fatty acids (SCFAs) by the strain, as well as other metabolites (Commane et al., 2005). The activity of the heat inactivated strain does however demonstrate that structural components of the BB-12 strain, such as cell wall components and extracellular proteins, could also play a role in improving barrier integrity in the GI tract (Castro-Herrera et al., 2020).

Several *in vivo* studies have also evaluated the impact of BB-12 supplementation on barrier function. In a DSS-induced model of colitis, eight-week-old male C57BL/6 J mice were first fed the BB-12 strain in fermented milk for 14 days and subsequently co-supplemented with 2.5% DSS for an additional week to induce colitis (Yan et al., 2020). Here, administration of the BB-12 strain in fermented milk was found to significantly improve the colonic histology scores of treated mice compared to those who received DSS alone. Supplementation with the BB-12 fermented milk was also found to significantly increase the relative expression of genes encoding the tight junction proteins zonulin and claudin, in addition to the expression of the MUC2 mucin encoding gene (Yan et al., 2020). The increase in the expression of these genes outlines how the BB-12 strain may help alleviate the damage to the mucosal barrier associated with the DSS challenge. In another model where BALB/c mice were injected with LPS to induce intestinal injury, supplementation with the BB-12 strain was shown to improve histological markers in the ileum. Here mice received 5E+09 CFU of the BB-12 strain daily for 14 days, whereafter they received an intraperitoneal injection of LPS (5 mg/kg) on day 14 and were sacrificed 6 h after LPS treatment. Here the villus height/crypt depth ratio was significantly greater in BB-12 supplemented mice compared to those treated with LPS alone, indicating improved intestinal barrier integrity and an improvement in the ability of the small intestine to absorb nutrients (Yue et al., 2023). A significant increase in the expression of the gene encoding MUC2 was again seen in this study, and although the expression of genes encoding tight junction proteins occludin, zonulin and claudin were increased, the changes were not found to be significant (Yue et al., 2023). While this study evaluates the efficacy of the BB-12 strain in preventing intestinal damage associated with an LPS challenge, it would have been interesting to track recovery from the LPS induced intestinal damage after additional supplementation of the strain.

In a long-term supplementation study where 8-month-old Crj: CD-1 mice were fed 1E+09 CFU/kg per dose of the BB-12 strain three times a week for 11 months, BB-12 was shown to improve barrier function, with lower levels of mucosal degradation seen in the colon compared to control mice (Matsumoto et al., 2011). In a lactulose/rhamnose intestinal permeability test, mice supplemented with BB-12 were shown to have a lower lactulose/rhamnose ratio in urine, indicating improved barrier function. The upregulation of the gene encoding MUC2 in response to BB-12 treatment was also evaluated in this study, however the increase did not reach statistical significance (*p* = 0.07). A significant increase in the expression of the gene encoding occludin was however found, outlining how barrier function could be improved through the upregulation of tight junctions in response to treatment with the strain (Matsumoto et al., 2011).

In a clinical trial in obese adults, the BB-12 strain was supplemented at a dose of 1E+09 CFU/day for three weeks alone or in combination with 5 g of prebiotic galactooligosaccharides (GOS) to identify a potential impact on intestinal barrier function (Krumbeck et al., 2018). Gastrointestinal barrier integrity was measured pre- and post-treatment following an aspirin challenge known to increase intestinal permeability. Participants ingested a sugar solution consisting of mannitol, lactulose, sucrose, and sucralose, and subsequent increases in urinary measurements of these sugars acted as markers of increased intestinal permeability. Supplementation with the BB-12 strain was shown to significantly reduce the excretion of sucralose in urine following the aspirin challenge, indicating that the BB-12 strain improves intestinal permeability in the colon. Interestingly, the markers of small intestinal permeability were unchanged, suggesting the activity of the strain is primarily seen in the colon (Krumbeck et al., 2018).

## Immune system

The human gut is home to a diverse array of immune cells, including dendritic cells (DCs), T cells, macrophages, monocytes, and B cells, each playing a crucial role in interacting with commensal bacteria, maintaining immune homeostasis and responding to pathogens (Lee, 2022). The impact of probiotics on the immune system has been shown to be strain specific where it is characterized by the recognition of cellular components by pattern recognition receptors (PRRs) present in host cells. Toll-like receptor 2 (TLR2) is the most extensively studied PRR involved in detecting Gram-positive bacteria such as BB-12. TLR2 recognizes bacterial membrane components including peptidoglycans and lipoteichoic acid. Upon antigen binding, it recruits myeloid differentiation primary response 88 (MyD88), initiating a signaling cascade that activates transcription factors nuclear factor-kappa B (NF-κB) and activator protein 1 (AP-1). This activation has been shown to induce cytokine secretion and immune cell maturation (Arenas-Padilla et al., 2022; Oliveira-Nascimento et al., 2012). Arenas-Padilla and colleagues demonstrated how IL-10 production from pig blood monocytes cocultured with BB-12 was mediated by TLR2 activation via cell wall components, whereas spent medium had no effect (Arenas-Padilla et al., 2022). Although TLR2 plays a critical role in BB-12 recognition, immune responses are likely not limited to a single receptor. In swine monocytes exposed to BB-12, for example, after TLR2 blockade micro RNA (miRNA) expression changes indicate that the strain could also be recognized by TLR9 (Arenas-Padilla et al., 2022). In support of this, another study showed how increased TLR9 gene expression was observed in human DCs after stimulation with BB-12 (Verbeek et al., 2010). This diversity of cellular PRRs and the potential of post-transcriptional regulation of immune responses induced by miRNAs might be one of the reasons why probiotic effects are strain dependent and context specific.

The maturation of DCs is of particular importance due to their crucial roles in antigen presentation and provision of co-stimulatory signals to T cells (Merad et al., 2013; Howard et al., 2004). The ability of the BB-12 strain to induce DC maturation has been well documented *in vitro*, with evidence demonstrating the upregulation of molecules such as Cluster of Differentiation (CD) 40, CD80, CD86, and Human Leukocyte Antigen—DR isotype (HLA-DR) (Verbeek et al., 2010; López et al., 2010; Latvala et al., 2008). Another key marker of immune cell maturation is the secretion of cytokines and chemokines, with multiple studies reporting increased levels of pro-inflammatory cytokines, including Interleukin (IL)-6, IL-1β, and Tumor Necrosis Factor (TNF)-*α* following BB-12 stimulation *in vitro* (Verbeek et al., 2010; López et al., 2010; Latvala et al., 2008). IL-6 is a pleiotropic cytokine involved in both pro-inflammatory and anti-inflammatory responses within the gut and promotes the differentiation of T and B cells, thus facilitating adaptive immune responses. Additionally, IL-6 plays a role in maintaining gut barrier integrity by influencing the production of mucins and antimicrobial peptides, shaping the gut microbiota composition and protecting the intestinal epithelium from pathogens (Hunter and Jones, 2015; Kuhn et al., 2018). Similarly, IL-1β contributes to gut homeostasis by regulating inflammation and immune cell recruitment. It also supports the differentiation and proliferation of T helper (T_H_)-17 and non-classically derived T_H_1 cells, both of which are essential for mucosal immunity (Turner et al., 2014; Akdis et al., 2016). TNF-*α* plays a dual role in inflammation, acting as a potent mediator of early immune responses while also limiting their extent and duration. Beyond its inflammatory functions, TNF-α regulates apoptosis, promotes cell proliferation, and activates various immune cells. Like IL-6, TNF-α also contributes to intestinal barrier integrity and gut microbiota modulation (Akdis et al., 2016; Trevejo et al., 2001).

Other key cytokines involved in gut health which are stimulated by the BB-12 strain include IL-12, interferon-gamma (IFN-*γ*), and IL-10 (Verbeek et al., 2010; López et al., 2010; Latvala et al., 2008; Arenas-Padilla et al., 2018). IL-12, primarily produced by activated DCs, is essential for the differentiation of naïve T cells into T_H_1 cells which are crucial for immune defence against intracellular pathogens. IL-12 also enhances the production of IFN-γ from T cells, which, in turn, activates macrophages and strengthens immune responses. Conversely, IL-10 serves as a critical regulatory cytokine that promotes the development of T regulatory cells (T_REG_), inhibits excessive inflammation, and fosters tolerance to commensal bacteria and dietary antigens (Schmitt and Ueno, 2015).

Secretion of chemokines such as IL-8, CXCL-10, and CCL20 has also been reported following the BB-12 strain stimulation (López et al., 2010; Latvala et al., 2008). Chemokines are responsible for directing the migration and arrest of immune cells during immune surveillance, homeostasis, and inflammatory responses (Chen et al., 2018). IL-8 primarily recruits neutrophils but also promotes the chemotactic migration and activation of monocytes, lymphocytes, basophils, and eosinophils, along with facilitating angiogenesis (Turner et al., 2014; Papadakis and Targan, 2000). CCL20, released in response to cytokine stimulation such as TNF or IL-1 via NF-κB activation, attracts memory T cells, immature DCs, and B cells to the mucosa. Similarly, CXCL-10, also known as Interferon gamma-induced protein 10 (IP-10), has been associated with the homing of activated lymphocytes (Wells et al., 2017; Hausmann et al., 2005).

Beyond its effects on DCs, the BB-12 strain has also been studied in the context of macrophage aging (Zhang et al., 2019). Immunosenescence, a state of dysregulated immune function, contributes to an increased incidence of chronic diseases, including infections, autoimmune disorders, chronic inflammatory diseases, and cancer (Wang et al., 2024). An *in vitro* study by Zhang et al. (2019) demonstrated that BB-12 attenuated macrophage aging induced by D-galactose. The study also showed that the strain significantly reduced the levels of pro-inflammatory cytokines IL-6 and IL-12 while increasing the expression of the anti-inflammatory cytokine IL-10 and the M2 polarization marker Arg-1 in J774A.1 macrophages. These findings suggest that BB-12 may enhance macrophage function by shifting the balance toward an anti-inflammatory phenotype, which is crucial for maintaining immune homeostasis and mitigating chronic inflammation (Zhang et al., 2019).

Animal models serve as invaluable tools for studying the immune effects of probiotics on gut health, providing a controlled environment to investigate the complex interactions between probiotics and the host immune system (Hoffmann et al., 2003). Using various animal models, the BB-12 strain has demonstrated immunomodulatory effects by enhancing mucosal immunity and modulating inflammatory responses. In gnotobiotic mice infected with *Salmonella,* BB-12 was shown to significantly increase intestinal secretory Immunoglobulin A (sIgA) levels (Martins et al., 2009). sIgA plays a crucial role in gut defence by binding to bacterial surfaces, and either trapping the microbe within the intestinal mucous layer or preventing their adhesion to epithelial cells, thereby inhibiting pathogen translocation (Cerutti et al., 2011). In a *Salmonella*-induced colitis model, BB-12 was shown to alleviate inflammation by modulating the relative expression of the colonic cytokines IL-1β, TNF-*α* and CXCL2, and reducing the secretion of IL-1β, IL-6, and TNF-α in serum and colonic tissues (Pang et al., 2021). Similarly, in a colitis model, BB-12 mitigated the LPS-induced epithelial barrier dysfunction by balancing pro- and anti-inflammatory cytokines, restoring mucus production inhibited by the LPS treatment, promoting IgA + plasma cells, and increasing the CD4+/CD8 + T cell ratio, which is a critical biomarker in assessing immune function (Yue et al., 2023; Lu et al., 2015).

Clinical studies investigating the effects of the BB-12 strain have further demonstrated its capacity to influence immune responses through multiple mechanisms. In a randomized crossover study, *ex vivo* stimulation of PBMCs from participants showed that BB-12 interacted with peripheral myeloid cells via TLR2. Blocking of TLR2 in PBMCs resulted in diminished TNF-*α* and IL-6 cytokine secretion upon BB-12 stimulation, further confirming its role in immune modulation. This study also highlighted an impact of the delivery matrix on immunomodulation, as participants of this trial who consumed yogurt smoothies with post-fermentation BB-12 addition, in comparison to pre-fermentation addition or dosing in capsules, exhibited lower TLR2 expression on CD14^+^HLA-DR^+^ cells and reduced TNF-α secretion after *ex vivo* stimulation with LPS or BB-12 (Meng et al., 2017).

In a vaccination study designed to evaluate the ability of the BB-12 strain to modulate the humoral immune response of healthy subjects, supplementation with the strain enhanced mucosal and systemic antibody responses to the vaccine. Notably, two vaccine-specific Immunoglobulin G (IgG) subclasses, IgG1 and IgG3, were significantly increased after probiotic supplementation, suggesting promotion of T_H_1 and T_H_2 lymphocytes activities. Similarly, significantly greater mean fold increases for vaccine-specific secretory IgA in saliva were observed in the probiotic group versus the placebo group. This enhancement of adaptive immune response to vaccination is considered to be associated with optimal protection against mucosal transmitted pathogens (Rizzardini et al., 2012). In a study of individuals undergoing a 10-day antibiotic regimen, BB-12 supplementation was shown to induce the upregulation of whole blood immune markers, including the transcription factor Interferon Regulatory Factor 8 (IRF-8) which regulates expression of genes stimulated by type I IFNs, the pattern recognition receptor TLR2, and the tumor necrosis factor receptor superfamily member 14 (TNFSF14) which mediates signal transduction pathways that activate the immune response. Additionally, BB-12 supplementation led to an increase in the CD80, CD40 and CXCL10 relative gene expression, indicative of immune activation and a potential T_H_1 polarization effect (Merenstein et al., 2015). Beyond its role in enhancing vaccine-induced immune responses and modulating immune markers during antibiotic use, BB-12 has also demonstrated protective effects against common infections. Ringel-Kulka et al. (2015) found that daily BB-12 supplementation in young children attending childcare significantly reduced the numbers of fever days and improved social and school functioning, suggesting immune-enhancing effects against common infections (Ringel-Kulka et al., 2015).

In summary, the immune-modulatory effects of BB-12 are well-supported by a robust body of evidence from *in vitro*, animal, and clinical studies, suggesting that the strain plays a pivotal role in immune regulation, offering broad benefits across different life stages and health conditions.

## Gut microbiota

**Figure 3:**
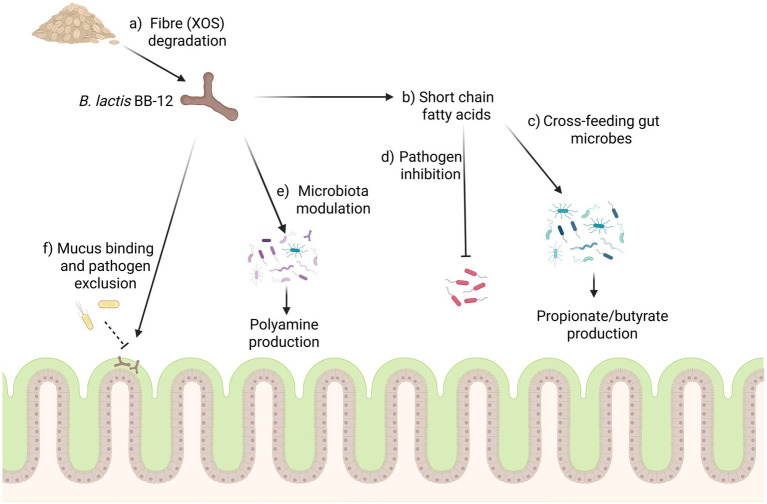
The growth of the BB-12 strain can have several potential impacts on the host microbiota, (a) with fibres such as XOS potentially acting as a prebiotic for the strain to grow. (b) The production of short chain fatty acids such as acetate and lactate can have several impacts on the microbiota, acting to cross-feed microbes in the GI tract leading to the production of compounds such as propionate and butyrate (c) and potentially inhibiting pathogenic microbes (d). (e) BB-12 may also modulate the microbiota to increase the synthesis of polyamines in the colon. (f) The strain has also been shown to bind to intestinal mucin where it can act to exclude pathogens from binding. Figure created in https://BioRender.com.

The gut microbiota has an important role in human health and disease, and the interaction of a probiotic with the host microbiota can form one mechanism by which a strain could impart health benefits (Chandrasekaran et al., 2024). This interaction between a probiotic bacterium and the host microbiota is multifaceted and may lead to a variety of ecological relationships between microbes (Culp and Goodman, 2023). Cross-feeding is one such example and involves sharing of resources between different microbes whereby metabolites released from one strain can be utilised by another to support its own growth (Culp and Goodman, 2023). This can be seen with the extracellular degradation of complex fibres by certain bacteria, which can lead to the release of simple sugars that can be utilised by surrounding microbes (Xiao et al., 2024). The SCFA acetate produced by bifidobacteria, for example, is an important intermediate in the production of butyrate by species such as *F. prausnitzii*, *Roseburia* spp., and *Eubacterium* spp. (Culp and Goodman, 2023). Butyrate has a functional role in the GI tract, acting as an energy source for colonocytes, which helps support barrier function and decrease inflammation. Increasing the butyrate concentration in the GI tract through cross-feeding between microbes is therefore considered a beneficial probiotic trait (Hodgkinson et al., 2023). Another mechanism by which probiotics can potentially impact the microbiota is through the inhibition of pathogenic microbes. This can occur through the release of inhibitory metabolites, such as organic acids or bacteriocins (Van Zyl et al., 2020). Additionally, strains can occupy nutritional niches in the environment which may have otherwise supported the growth of pathogens, thus inhibiting their establishment by competitive exclusion (Chandrasekaran et al., 2024). This inhibition of growth and colonization of pathogens demonstrates mechanisms by which probiotic microbes can increase the resilience of the host microbiota and help to reduce the risk of gastrointestinal disturbances (Mills et al., 2018; Liu et al., 2022). A range of these interactions between a probiotic and the gut microbiota have been studied and demonstrated with the BB-12 strain (Figure 3).

The interaction between the BB-12 strain and potentially pathogenic microbes in the GI tract has been assessed in several studies. In *in vitro* assays, BB-12 has been shown to reduce binding of a wide variety of pathogens to intestinal mucus through competitive exclusion. The strain was also shown to displace pathogenic bacteria already bound to mucus in these assays (Collado et al., 2007a; Collado et al., 2007b). Further, metabolites produced by BB-12 such as organic acids have been shown to inhibit a range of pathogens such as *Clostridioides difficile* and *L. monocytogenes* (Martins et al., 2009; Kondepudi et al., 2012). In a mouse model of *Salmonella Typhimurium* infection, supplementation with BB-12 was shown to reduce the colonization of the pathogen as well as reversing some of the symptoms associated with infection (Pang et al., 2021). Analysis of the SCFAs in the caecum of these mice found that while lactate and acetate levels were unchanged after BB-12 supplementation, there was an increase in propionate levels. This suggests that BB-12 was modulating the microbiota, potentially through cross-feeding, to increase propionate production (Pang et al., 2021). Propionate was found to have a strong inhibitory effect on *Salmonella* growth *in vitro*, thus demonstrating how the interaction of BB-12 with the host microbiota can help reduce pathogen colonization (Pang et al., 2021). In the absence of an established host microbiota the impact of BB-12 was found to be more subtle, as was seen in germ-free pig models of *Salmonella Typhimurium* infection (Splichalova et al., 2021; Splichal et al., 2023). In these studies, BB-12 was found to reduce *Salmonella* load and improve immune markers, however, it would be interesting to understand if the interactions between BB-12 and an existing gut microbiota could have had a greater impact on pathogen colonization (Splichalova et al., 2021; Splichal et al., 2023).

Through the metabolism of dietary fibres, the BB-12 strain can also impact the microbiota via the production of SCFAs which can potentially cross-feed other microbes in the gut. One example of this was seen with dietary xylo-oligosaccharides (XOS), which are composed of chains of xylose residues produced industrially through the partial hydrolysis of plant hemicelluloses (Gilad et al., 2010; Zhao et al., 2024). XOS cannot be digested by humans and supplementation has been associated with increased levels of lactobacilli and bifidobacteria in the GI tract, suggesting XOS could function as a targeted prebiotic (Pang et al., 2021; Capetti et al., 2021). Research on BB-12 demonstrates that the strain has the ability to bind XOS at the surface of the cell, import the sugar, and metabolize it internally (Gilad et al., 2010). The targeted growth of the BB-12 strain can lead to an increase in the production of acetate and lactate, which in turn can cross-feed other microbes, potentially leading to an increase in propionate and butyrate synthesis (Pang et al., 2021; Rios-Covian et al., 2015).

The cross-feeding between the BB-12 strain and members of the gut microbiota has also been identified through the production of polyamines. Polyamines are organic molecules composed of two or more amino groups and play critical structural and functional roles in both bacterial and eukaryotic cells. These are essential for cell growth and proliferation, with spermine, spermidine and putrescine being the primary polyamines found in human cells (Muñoz-Esparza et al., 2019). Polyamine levels have been associated with longevity in animal models, and changing levels in humans have been associated with aging and disease (Nishiwaki et al., 2024; Minois et al., 2011). Polyamines can be produced naturally in the body, with diet also being a major source, and when ingested, these polyamines are typically absorbed in the duodenum (Muñoz-Esparza et al., 2019). Polyamines found in the large intestine are, however, typically synthesized by microbes in the lower GI tract and can be transported to the blood stream via the colonic mucosa (Matsumoto and Benno, 2007; Tofalo et al., 2019).

Long-term supplementation with the BB-12 strain was shown to increase the concentration of faecal polyamines in a variety of mouse models (Matsumoto et al., 2011; Kibe et al., 2014; Ma et al., 2022). This increase in the concentration of polyamines in response to BB-12 supplementation has also been noted in several human studies, however, there have been instances where no increase in polyamines has been identified (Matsumoto et al., 2007; Matsumoto and Benno, 2004; Matsumoto et al., 2001; Manzoni et al., 2017). The primary means by which BB-12 influences polyamine levels in the gut is likely through the modulation of the intestinal microbiota (Tofalo et al., 2019; Kitada et al., 2018). In preclinical models, it has been shown that the acidification of intestinal conditions by BB-12 through the production of SCFAs induced a stress response in gut microbes which leads to the increased synthesis and export of putrescine via the utilization and synthesis of arginine and agmatine, a trait which did not appear to be universal across the other species of bifidobacteria tested (Kitada et al., 2018). Supplementation of the strain alongside polyamine precursors such as arginine may be one approach to help boost polyamine production further (Kibe et al., 2014).

The ability of the strain to impact markers of health through the modulation of the microbiota was also identified in a study by Matsumoto et al. (2017). In this randomized, double blind placebo controlled clinical trial, the impact of supplementation with the BB-12 strain on risk factors for atherosclerosis was evaluated. Here the strain was shown to significantly reduce faecal trimethylamine (TMA) levels, a precursor to the pro-atherogenic metabolite trimethylamine N-oxide (TMAO) which can contribute to the formation of vascular plaques. This reduction was likely a result of the modulation in the composition of bacteria in the gut, as seen by a decreased prevalence of TMA-producing Clostridiales and Lachnospiraceae (Matsumoto et al., 2017). There is also a strong link between TNF-*α* levels and atherosclerosis, and the impact of BB-12 in reducing such inflammatory cytokines further supports its benefits in cardiovascular health (Matsumoto et al., 2017; Nanami et al., 2005).

The BB-12 strain has also been shown to have a positive impact on the microbiota of individuals who have recently received antibiotic treatment (Merenstein et al., 2021). In a human clinical trial, participants were treated with antibiotics for 7 days, while also receiving either a control yoghurt or yoghurt containing the BB-12 strain. Supplementation with BB-12 or control yoghurts extended an additional 7 days after antibiotic treatment ceased, and this was followed by a two-week washout period (Merenstein et al., 2021). By measuring the faecal SCFA concentrations post antibiotic supplementation, it was seen that individuals supplemented with the BB-12 yoghurt had a faster restoration of faecal acetate levels to baseline compared to controls. It was also seen that BB-12 supplementation attenuated the reduction in propionate and butyrate levels following antibiotic treatment. As these SCFAs are not directly synthesized by BB-12, it was hypothesized that the strain helps boost these levels via modulation of the host microbiota (Merenstein et al., 2021). Through faecal microbiome analysis it was shown that BB-12 supplementation reduced the impact on microbial diversity and helped the microbiome in the gut recover at a faster rate compared to controls after antibiotic treatment (Merenstein et al., 2021). The production of acetate acidifies the intestinal environment, which is less favorable to potential pathogens and provides more suitable conditions for commensal microbes (Merenstein et al., 2021). The acetate produced by the BB-12 strain can also act to cross-feed other microbes and support butyrogenic bacteria, which could explain the attenuated decrease in butyrate levels associated with BB-12 supplementation after antibiotic treatment (Culp and Goodman, 2023; Merenstein et al., 2021). In clinical studies in infants experiencing colic, supplementation with BB-12 was shown to increase levels of faecal butyrate, most likely as a result of cross-feeding with other microbes (Nocerino et al., 2020; Chen et al., 2021). The impact of BB-12 on faecal SCFA levels was evaluated in separate studies in a healthy population and in individuals experiencing atopic dermatitis, however, consistent increases in SCFAs were not identified across these studies (Lee et al., 2017; Matsumoto et al., 2007).

## Low defecation frequency

In several clinical trials, the BB-12 strain has demonstrated an ability to improve symptoms in individuals experiencing a low frequency of defecation. The pathophysiology of slower GI transit and constipation is complex and multifaceted, and involves aspects of hormonal balance, the nervous and immune systems, gut ecology and many other factors (Araújo and Botelho, 2022). In a double-blind crossover trial in healthy females, individuals were supplemented with a BB-12 fermented milk (1E+09 CFU/100 g) or a control fermented milk for 2 weeks, with a 2-week washout period included between treatments (Uchida et al., 2005). Supplementation with BB-12 was found to significantly increase stool frequency in the population in comparison to the control intake period, and this result appeared to be driven by an improvement observed in those with a tendency for constipation (Uchida et al., 2005). Another crossover trial was carried out with BB-12 fermented milk, with a reduced treatment and washout period of only 1 week, and a lower dose of BB-12 (at least 1E+08 CFU/100 g). In this trial no significant difference in stool frequency was seen between the groups receiving BB-12 and control fermented milks, respectively. This suggests that the lower dose used, or the shorter timeframe of supplementation, may not have been sufficient to show improvements in symptoms (Uchida et al., 2004). In this second study, the number of participants showing a tendency for constipation (*n =* 12) was lower compared to the first (*n =* 29) and the reduced sample size of this key subpopulation may also have influenced the ability to detect significant differences.

In a large, multi-centre, double-blind placebo controlled clinical trial with over 1,200 participants, the BB-12 strain was supplemented at two different doses (1E+09 CFU/day and 1E+10 CFU/day) for 4 weeks in individuals with a low frequency of defecation and abdominal discomfort (Eskesen et al., 2015). When defining a responder as an individual with an increase from baseline of at least one complete spontaneous bowel movement per week, treatment with BB-12 did not reach significance over placebo given the strong placebo effect seen in the study. This was despite the fact that individuals supplemented with BB-12 had an average defecation frequency significantly higher than the placebo at all weeks (Eskesen et al., 2015). When applying more strict responder criteria by defining a responder as a subject with an increase in defecation frequency from baseline of at least 1 defecation/week for at least 50% of the time, a significant improvement was seen in individuals supplemented with BB-12 (Eskesen et al., 2015). Supplementation with both concentrations of BB-12 was found to have a similar effect, suggesting efficacy can be achieved with a dose of 1E+09 CFU/day of BB-12 (Eskesen et al., 2015).

In a long-term study in elderly nursing home residents, the impact of the BB-12 strain on normalizing bowel movements was evaluated (Pitkälä et al., 2007). In this study, BB-12 was supplemented in the form of a fermented oat drink at a dose of 1E+09 CFU/day for 7 months, with a pasteurized oat drink used as a control. The regularity of bowel movements and the consistency of stools were then measured throughout the study in participants to determine the impact of the strain on bowel function. Here, the BB-12 fermented oat drink was shown to have a significant effect in normalizing participant’s bowel function, significantly increasing the frequency of bowel movements in subjects. This demonstrates the efficacy of the BB-12 fermented oat drink in alleviating the effects of a low frequency of defecation in an elderly population.

Given a range of influences can impact gut motility, there are several mechanisms by which the BB-12 strain could have this impact with the modulation of SCFAs in the gut by the strain being one such mechanism. It has been shown that faecal acetic and propionic acid levels are significantly lower in individuals experiencing slow transit constipation compared to healthy controls, while isobutyric and isovaleric acid levels are higher (Chen et al., 2024). SCFAs have been shown to bind to G-protein-coupled receptors on enterocytes which can impact the contraction frequency of the colon and thus affect GI motility. How SCFAs impact colonic motility through these receptors appears to be highly conditional, and dependent on the balance of the SCFA composition and levels in the gut (Jiang et al., 2022). Understanding the impact which BB-12 has directly, or indirectly through cross-feeding, on intestinal SCFA levels and how this affects GI motility may help elucidate the mechanism by which the strain can improve symptoms in individuals experiencing a low frequency of defecation.

## Colic

The BB-12 strain has been shown to help alleviate symptoms associated with colic in infants (Nocerino et al., 2020; Chen et al., 2021). According to the Rome IV criteria, infant colic is defined as recurrent and prolonged periods of fussing/crying/irritability which cannot be resolved by caregivers in infants from birth to 5 months of age with no evidence of fever or illness or a failure to thrive (Muhardi et al., 2022). It is a relatively common phenomenon, and while self-limiting, it can be a source of distress for the infants and have a major impact on the quality of life of parents and caregivers (Zeevenhooven et al., 2017). The pathophysiology of colic is complex and unclear, however it likely involves the interaction of a number of elements such as gastrointestinal, neurodevelopmental and immune factors, while also being impacted by microbiome composition and activity, mode of feeding and psychosocial influences (Zeevenhooven et al., 2018). To evaluate the potential impact which BB-12 may have on colic, two separate randomized clinical trials were carried out in infants.

The first trial by Nocerino et al. (2020) was a double-blind, randomized, placebo-controlled trial in 80 infants. Exclusively breastfed infants aged ≤7 weeks who had signs and symptoms related to infant colic according to Rome III criteria were recruited. Infants were supplemented with either BB-12 oil drops (1E+09 CFU/day) or placebo oil drops for 28 days. Supplementation with BB-12 resulted in a significantly higher reduction in crying duration over the treatment period compared to the placebo group, with the mean daily duration of crying episodes found to be consistently shorter in the BB-12 group compared to placebo. There was also a significantly greater reduction in mean number of crying episodes compared to baseline in infants supplemented with BB-12 than those in the placebo group. Analysis of faecal samples identified an increase in the concentration of butyrate in BB-12 treated infants, likely associated with cross-feeding between the strain and the existing microbiota of these infants. An evaluation of immune markers also identified a significant increase in HBD-2, LL-37 and sIgA levels in infants receiving BB-12 compared to those receiving placebo, suggesting an immunomodulatory impact associated with BB-12 supplementation (Nocerino et al., 2020).

A subsequent double-blind, randomized, placebo-controlled trial was carried out in 192 full-term infants meeting the Rome III criteria for colic in Chengdu, China (Chen et al., 2021). Exclusively breastfed infants were enrolled in this study and were <12 weeks of age at time of enrolment. After a 1 week run in period, infants were supplemented with either BB-12 oil drops (1E+09 CFU/day) or placebo oil drops for 21 days. Supplementation with BB-12 was shown to significantly increase the percentage of infants with a reduction in daily crying/fussing time by ≥50% after 21 days of supplementation compared to placebo. BB-12 supplementation again led to a significant reduction in mean number of daily crying episodes and in duration of crying time compared to placebo supplemented infants. There was also a significant increase in daily sleep duration in infants receiving BB-12 compared to those receiving the placebo. As seen in the previous study, faecal levels of butyrate were found to be significantly increased compared to placebo, as were HBD-2, LL-37 and sIgA levels. Given the impact which infant colic can have on parents and caregivers, a modified version of the PedsQL module was used to measure the parents’/caregivers’ health related quality of life (HRQoL). It was found that parents/caregivers of infants in the BB-12 supplemented group had significantly improved scores compared to those of placebo treated infants, suggesting that the improvement of colic related symptoms in infants receiving BB-12 were associated with an improved quality of life in their caregivers (Chen et al., 2021).

While the exact mechanisms of action of the BB-12 strain on infant colic are still to be elucidated, there are a number of contributing factors which BB-12 may target. The composition of the microbiota of infants is one such factor which appears to contribute to infant colic (Zeevenhooven et al., 2018). It has been seen that the microbiome of infants with colic contains a lower proportion of bifidobacteria compared to control infants (Rhoads et al., 2018; Kozhakhmetov et al., 2023). In the Nocerino et al. (2020) study, infants defined as responders who were supplemented with BB-12 were found to have a significant increase in bifidobacteria, indicating a shift from a microbiome profile associated with colic (Nocerino et al., 2020). It has been shown that the microbiome of infants with colic can also contain a greater level of gas producing microbes, with excess gas production potentially resulting in discomfort in infants that can lead to colic like symptoms (Ouald Chaib et al., 2020; De Weerth et al., 2013). By increasing the levels of bifidobacteria, BB-12 supplementation could potentially act to reduce the impact of those colic associated microbes (Nocerino et al., 2020). The levels of butyrate-producing bacteria have also been identified as being lower in infants experiencing colic (De Weerth et al., 2013). As previously mentioned, in both clinical trials involving supplementation with BB-12, significantly increased levels of faecal butyrate were found, likely through cross-feeding of other microbes (Nocerino et al., 2020; Chen et al., 2021). This suggests that BB-12 supplementation may mitigate some of the impacts linked to imbalances in the microbiome which are associated with colic.

## Conclusion

The mechanisms underlying the probiotic efficacy of the BB-12 strain are complex and multi-faceted and are likely mediated by interactions directly with the host as well as indirectly via the microbiota. Given the extent of the research carried out on the BB-12 strain, there is a unique opportunity to study a broad range of aspects of probiotic-host interaction. Here we outline the ability of the strain to survive in the GI tract, overcoming the acidic conditions of the gut as well as the antimicrobial activity of bile acids. This is seen in both preclinical experiments and clinical trials where the strain is isolated from the faecal samples of participants, indicating live transit of BB-12. The establishment of BB-12 in the host gut microbiota does appear to be transient, with colonization being diminished after supplementation with the strain has ceased.

The BB-12 strain has also demonstrated the ability to interact directly with host cells, including gut epithelial cells and host immune cells. Evidence of this can be seen in a clinical setting where BB-12 has been shown to improve colonic barrier function (Krumbeck et al., 2018). The immune-modulatory effects of BB-12 are also well-supported by a robust body of evidence from *in vitro*, animal, and clinical studies. *In vitro* data highlights the strain’s role in immune cell maturation leading to the secretion of key immune modulatory cytokines and chemokines, and induction of T_H_1 responses. Animal models demonstrate the strain’s ability to enhance IgA secretion during infections and reduce inflammation in response to diverse inflammatory challenges, with clinical studies further validating such findings. The strain’s impact is evident not only on host cells and tissues, but also on the existing host gut microbiota. This has been demonstrated across multiple studies where the strain was shown to increase the levels of key SCFAs such as butyrate, likely as a result of cross-feeding with other gut microbes. This influence on the gut microbiome can also be seen with the effect BB-12 has on reducing the impacts of antibiotic treatment (Merenstein et al., 2021). Focusing on key health conditions, supplementation with BB-12 has been shown to improve the frequency of defecation at doses of 1E+09 and 1E+10 CFU/day in individuals with a low defecation frequency and abdominal discomfort (Eskesen et al., 2015). In children suffering from infant colic, BB-12 has been shown to improve key symptoms such as the number and duration of crying episodes. This improvement in symptoms was also associated with an improved quality of life for the caregivers for these infants (Nocerino et al., 2020; Chen et al., 2021).

Despite being one of the most extensively studied probiotic strains on the market, further trials could benefit from including endpoints aimed at substantiating the mechanisms of action behind the effects associated with BB-12 supplementation. Additional preclinical studies could also allow researchers to elucidate some of these mechanisms of action, with a focus on both the direct and indirect impact of the strain on its environment. Uncovering some of these mechanisms may not only allow us to gain a greater understanding of the currently studied effects of the strain, but also uncover novel health targets where the strain could demonstrate benefits.

Taken together, the data reviewed in this study demonstrates the efficacy of the BB-12 strain in surviving the harsh conditions of the GI tract, as well as outlining the range of health benefits associated with supplementation of the strain. These benefits, as well as the established safety and application versatility of the strain, establish why BB-12 is considered one of the more prominent probiotic strains currently on the market.
